# From Self-Transcendence to Collective Transcendence: In Search of the Order of Hierarchies in Maslow’s Transcendence

**DOI:** 10.3389/fpsyg.2022.787591

**Published:** 2022-03-24

**Authors:** Luis Felipe Llanos, Lorena Martínez Verduzco

**Affiliations:** Business and Economics School, Anáhuac University, Naucalpan de Juárez, Mexico

**Keywords:** self-actualization, individual transcendence, collective transcendence, self-transcendence scale, human motivation

## Abstract

Maslow’s Human Motivation extended Theory, in its late version, proposed transcendence as one of the highest levels, inclusive or holistic in the Human consciousness. Through Meaning Theory, Victor Frankl and Paul Wong suggested that self-transcendence is a fundamental expression of our spiritual nature and a distinctive concept. However, it is not clear whether at present, with an extensive offer of individualistic currents, transcending involves a personal issue or is rather a collective issue, related to community and culture. The objective of this research is to determine if seeking personal transcendence and seeking collective transcendence are two differentiable categories, and if there is a priority among them, which would suggest an extension of the Theory. With the participation of 402 business students in Mexico, and with the help of a structured equation model, it was determined that there is a significant difference between orientation toward “I transcend” and the orientation toward “I help others transcend.” The results obtained from the selected sample indicate that individual transcendence and collective transcendence are distinguishable categories. In addition, it was found that young people prefer to seek a personal meaning rather than a collective one, which suggest that dimension of collective transcendence is prior to personal transcendence. These findings can be used to design job profiles for positions that require staff development, and it can serve as a positive pattern for life education and society.

## Introduction

The discussion about the social benefits between individualism and collectivism is not over yet (Černe et al., 2013; Valaei et al., 2016; Raza et al., 2018). Heidegger (1977, p. 128) identifies that as a consequence of the liberation of the human being, subjectivism and individualism have been introduced, “but never like before, has the collective come to be accepted as valuable.” Twenge et al. (2012) comment in their book Generation Me vs. Generation Us, that competitiveness and technological change are leading young people to be self-sufficient and to be less concerned about others compared to previous generations. The preference between individualism and collectivism has implications on people’s motivation and well-being, and differs according to culture. An example of this is the study by Leibbrandt et al. (2013), they found that the most competitive fishers belong to individualistic societies, while the least competitive ones belong to collectivist societies.

Santos et al. (2017) examined 51 years of information on individualistic values and practices in 78 countries; they found that individualism is on the rise in most societies; however, depending on different cultures, it advances at different speeds. Countries with lower socioeconomic development show a subtle increase in their individualistic values, while in countries with higher socioeconomic development, individualism increases faster. These same authors found that autonomous practices are increasing in most societies, so it seems that human beings need to assume individualistic and materialistic values to adapt to urban life (Greenfield, 2013). Markus and Kitayama (1991) proposed to measure the contrasts between the independence and interdependence constructs in different cultures, without great results. However, in the past decade, and particularly from the in-depth performance of two international studies (see Becker et al., 2012; Becker et al., 2014), it can be concluded that there are distinctive cultural traits that motivate people to be independent or interdependent. Among the distinctive features, collectivism, national socioeconomic development and religious heritage were identified (Vignoles et al., 2016). However, Hofstede (2001, p. 19) exposes the following caveat: “cultures are not giant individuals” and their internal logic cannot be understood in the terms used for the dynamics of the personality of individuals.

### Self-Actualization

Maslow (1943) has already identified five basic human needs: the physiological needs, the safety needs, the love needs, the esteem needs, and the need for self-actualization, in this hierarchical order. The last step defines it as a tendency of the human being to achieve what he can potentially be. “This tendency could be expressed as the desire to become more and more what one is, to become all that one is capable of becoming” (p. 382). Those who achieve self-actualization, the author calls “satisfied people,” this last step is also known as self-improvement (Castro-Molina, 2018). However, in Maslow’s own words (2007, p. 141, no. 24) “in practice it happens very seldom, according to my criteria, in less than 1% of the adult population.” Castro-Molina (2018) proposes to see Maslow’s self-realization as an ideal that every human being wishes to reach and states that, during his journey, he develops his talent and potential by expressing his ideas and knowledge, which makes him grow and become a great person through the significance of his own work. Maslow (2007) himself, when delving into his theory, proposes that self-actualized people are more capable of enjoying (p. 143, no. 30) and loving (p. 145, no. 34). Furthermore, Maslow (2007, p. 137, no. 14.) specifies that “the mental health of an adult has several names: self-actualization, emotional maturity, individuation, productivity, authenticity, integral humanity.”

Boeree (2006), when writing the biography of Maslow, narrates that his methodology is the critical point of the hierarchy of self-actualization, which was not exhaustive, since for his analysis he only chose a small group of people who he himself declared as self-actualizing. Boeree (2006) also disqualifies self-actualization as a theory, since it is based on an exception, which reaches a minimum percentage of the human species, including artists, scientists and literary authors, who despite their shortcomings, poverty, bad education, neurosis and depression, reached self-actualization (e.g., Galileo, Rembrandt, Toulouse-Lautrec, Van Gogh, Trachtenberg or Viktor Frankl).

Boeree (2006), in defense of Maslow, explains that the same New York psychologist expected other researchers to take up the cause and complete what he had started in a more rigorous way. Alderfer (1969), proposed to strengthen Abraham Maslow’s idea by categorizing the five needs into three: (a) existence needs, (b) affinity, and (c) growth. In contrast, he proposes that the satisfaction of a lower level is not a prerequisite for the emergence of higher-order needs. Subsequently, following the line of Wahba and Bridwell (1976), reviewed 13 studies on the subject and added to the idea that there was little evidence that Maslow could present their needs in a hierarchical way, and with a determined order. They corroborated that the self-actualization factor “cannot be a basic need” (p. 233), but rather, as Berkowitz (1969) pointed out, “it is a desire for how a man would like to be” (p. 87).

Mwanzia (2009) explains that hierarchical needs do help to understand development processes, but they fall short to understand levels of motivation. In this way, Neef et al. (2010, p. 21), proposed, “perhaps it is more appropriate to speak of living and solving needs in a continuous and renewed way.” They suggest that the system of human needs operates through compensation and exchanges between each of the three fundamental subsystems: (a) human, individual and collective needs and potentials, (b) satisfactors, of an individual and collective nature as well, (c) economic goods. In other words, the three subsystems interact and subsidize each other. In turn, Koltko-Rivera (2006) takes up the concept of transcendence, and argues that self-actualization is not the highest hierarchical level, but rather a search for self-transcendence, which allows a better understanding of the meaning of life, well-being and motivation.

Maslow proposes that in the hierarchy of self-realization, many dichotomies are resolved, and that it is not convenient to divide it in two, the interior and the exterior, or between the self and the others. Maslow himself (2007, p. 143, no. 30) points out, “People who self-actualize have a strong tendency to merge selfishness and altruism in a superior, superordinate unit”; furthermore, he remarks that a symptom of an immature personality is if the hierarchy is divided. He also states that: “dichotomization currently appears to be a characteristic of a lower level of personality development and psychic functioning; it is both a cause and an effect of a psychopathology” (p. 143, no. 30).

In this sense, the first critic of this last hierarchy is Maslow himself (1943, p. 383), who recognized that self-actualization “continues to be a challenging problem for research.”

### Self-Trascendence

Maslow (1969b) addresses the issue of transcendence as an extension of his first theory of hierarchies, explains that transcendence is an end that is only reached in the complete hierarchical integration (p. 62), “Trascendence refers to the very highest and most inclusive or holistic levels of human consciousness, behaving and relating, as ends rather than means, to oneself, to significant others, to human beings in general” (p. 66).

Koltko-Rivera (2006, p. 302) suggests that, by dividing the concepts self-actualization and self-Transcendence, it allows a better understanding of the meaning of life, well-being and motivation, in particular “broader understanding of the motivational roots of altruism, social progress, and wisdom.” Studying Maslow’s (1943, 1954) conventional description of the hierarchy of needs, he proposes that “is inaccurate as a description of Maslow’s later thought. Maslow (1969a) amended his model, placing self-transcendence as a motivational step beyond self –actualization” (p. 302).

Starck (2014, p. 93) in his chapter Theory of Meaning, specifically clarifies that “the transcendence of Maslow (1968) should not be confused with the transcendence of Frankl (1975), since both start from different approaches. For Frankl (1963, p. 112), transcendence is a permanent state of the human being; he said “and ultimately, man transcends himself; the human being is a self-transcendent being” (p. 130). Recent studies highlight the idea that, by participating in rituals and collective meetings, where there are emotional exchanges and the convergence of ideals, subjective and psychological well-being, the meaning of life is reinforced and a collective sense of transcendent values is generated (Zumeta et al., 2020). Furthermore, the existence of a sense of transnational transcendence is proposed, in which, through discourse, it is sought to overcome personal interests in the short term in the search for a greater good, and collective well-being in the long term.

Also, Frankl (1954) himself considered self-actualization a by-product of self-transcendence, and introduced interpersonal dimensions “It denotes the fact that being human always points, and is directed, to something or someone, other than oneself—be it a meaning to fulfill or another human being to encounter” (Frankl, 1985, p. 133).

### Interpersonal Dimensions of Self-Trascendence

Many theorists, i.e., Maslow, Tornstam, Frankl, and Reed, have proposed subdividing self-transcendence into different dimensions, including the interpersonal or collective dimension.

In his study, Maslow (1969b) analyzes the transcendence from several perspectives, transcendence of time, space, culture, ego (of self), opinion (opinion of others), dichotomy, negative, and identification-love. He explains about this last dimension: “This means unselfish. This means transcendence of the selfish Self. It also implies a wider circle of identifications, i.e., with more people approaching the limit of identification with all human beings” (p. 59).

Tornstam (2003, 2005, 2011), delves into the study of transcendence during the different stages of life, explains that this occurs in three dimensions, the cosmic dimension, the self-dimension and the social and personal relationship dimensions, in the latter he proposes the importance of social contacts, social masks, emancipated innocence, reduced interest in material goods and wisdom, under a dimension that he called social and personal relationships.

Likewise, Reed (2009, p. 111), defines self-transendence as “a characteristic of developmental maturity in terms of an enhanced awareness of the environment and an orientation toward broadened perspectives about life. It is expressed and measured through life perspectives and behaviors that represent this pandimensional expansion of boundaries.” She managed to factor it into 4 dimensions: intrapersonal, temporal, transpersonal, and interpersonal, in particular, on this last dimension, it encompasses the relationship with others and with one’s environment.

Wong (2016), based on Frankl’s studies, noticed that seeking for the meaning of self-transcendence is based on our spiritual values of compassion, conscience, and serving others. In particular, he presents empirical evidence supporting the hypothesis that the search for meaning is healthier than the search for personal happiness.

### Collective Trascendence

Organizational cultures, in which self-questioning, balance, wisdom and reflection are valued and appreciated, people need to go beyond themselves to reach human potential. Transcendence is intrinsically related with the drive to develop a belonging and compassionate culture; which leads us to the dynamics of connectivity (Karakas et al., 2015).

Currently, there is a strong economic tendency to generate individual solutions to collective problems (Grossmann and Varnum, 2015), without necessarily still producing the required benefits. For example, with private education (Bernal and Lorenzo, 2013), insurance for major medical expenses (Homedes and Ugalde, 2002) or voluntary savings for self-retirement (Ortiz et al., 2019). Businesses also show a notable tendency to target market niches with individual products and solutions, suggesting different cost-benefit relationships to individually solve social needs (Gross and De Dreu, 2019). More than 100 years ago, many problems were solved with collective solutions, hence, for example, the beginning of the actuarial colleges (Simon, 1988). However, the world has taken another course, individualism, which undoubtedly leads to inefficient resource allocations and coordination failures, as analyzed in depth by Gross and De Dreu (2019) in their study of the “modern tragedy of the commons.”

Maslow discussed both, the relational unity of transcendence (collective) and the interpersonal/transpersonal (egoless/spiritual). The problem is that his amended theory has not been incorporated into textbooks, but he definitely discussed self-transcendence as the highest level of human consciousness, and it was Maslow who introduced “transhumanistic psychology” which he later called “transpersonal psychology” (Maslow, 1969b; Koltko-Rivera, 1998).

Thus, the study of transcendence from a personal and collective point of view is proposed aligned with the dimension of Identification-love proposed by Maslow (1969b, p. 59): “This means transcendence of the selfish Self,” more than with the interpersonal dimensions of Reed (2009), and social dimensions of Tornstam (2003, 2005, 2011), since in their definitions, the transcendence of the self predominates.

Heidegger (2007, p. 190), points out that transcending is a relational concept (of the subjects), where the transcendent is that toward which the step is taken, and what transcends (the object) is that which performs the step beyond (p. 188), in particular, for our research, the subjects will be the people interviewed, and as objects to be transcended. Two scenarios will be presented, the same subjects in a personal vision and in a collective vision.

Recently, Wong (2016, 317) named one of the levels of self transcendence “Seeking one’s calling,” in terms of reaching beyond self-actualization and pursuing a higher purpose for the greater good. This involves engagement and striving to achieve a concrete meaning in life, a life goal of contributing with something valuable to others.

In this research, we will focus in the latter dimension of collective transcendence and define as the concern of an individual calling that transcends his immediate community, in the sense that those close to him enjoy their life, accept themselves, find meaning in their existence and achieve their dreams, and ultimately that his community helps other communities to transcend, in a virtuous circle.

Therefore, the present investigation seeks to contribute to the debate on the following questions: does the orientation toward a personal transcendence differ from the orientation toward a collective transcendence? And if this is the case, do young people prefer to seek a personal transcendence rather than a collective one?

The discussion about the convenience between individualism or collectivism is still in place (Černe et al., 2013; Valaei et al., 2016; Raza et al., 2018). Consequently, we believe that it is pertinent to study whether the two orientations of transcendence can be significantly distinguished, the individualistic “I transcend” or the collectivist “I help others transcend.”

In line with the different theses that the human being is a self-transcendent being (Frankl, 1963) and that transcendence refers to the highest levels of human consciousness (Maslow, 1969b), the following research hypotheses are proposed: H1: The orientation toward personal transcendence and the orientation toward collective transcendence are two differentiable categories. H2: Young people prefer to seek a personal trascendence rather than a collective one.

## Materials and Methods

### Participants and Procedure

402 young students between 18 and 22 years old, of both genders, were sampled between August 29, 2019 and September 10, 2019. The students were studying a career in Business (Administration, Economics, Finance, and Accounting, Marketing or International Business). The university is private in Mexico, and charges tuition fees, so most of the students come from upper-middle-income families in the metropolitan area of Mexico City. University professors explained the questionnaire objective to the students. Students were asked to answer the questionnaire honestly and voluntarily in class. The questionnaire was electronic in order to randomly modify the order of the questions to each respondent. In order to guarantee anonymity, it was decided not to ask for any demographic data.

### Instruments

There are several proposals for validated instruments to measure self-transcendence, e.g., STM-B by Wong et al. (2021) and STS by Reed (2009), which contain interpersonal or collective questions, from the perspective of the Self. However, in its original versions, there is no dimension of collective transcendence such as the one that seeks to study the orientation of an individual toward the transcendence of his immediate community, from the perspective of the Other. Therefore, the strategy of adapting a current instrument and giving it a collective approach was adopted.

The STS scale of Reed (2009, 2014) was adapted to measure the orientation toward collective transcendence. This scale has been used in most research on this topic. The STS has demonstrated reliability and construct validity, and is short and easy to administer as a questionnaire.

Now, in regards to using the STS scale in a different language, the Norwegian research by Haugan et al. (2012), identified some problems in the literal translation of the language, e.g., item 15 “Letting go of my past losses,” since it seemed to exist by itself, constituting an uncorrelated factor, interpreted as the temporal aspect. Likewise, the Colombian research by Pena-Gayo et al. (2018), also found that the syntactic construction of items 2, 4, 5, and 15 in a literal translation was not relevant; the reason why they proposed to include more natural wording, syntactic corrections, inclusive language, a better definition of the concepts, which it obtained a satisfactory validation.

The translation and adaptation of the STS required generating two versions, one for each of the 15 items. A first version was written in the first person, where the one that transcended was the respondent himself; and a second version, where the previous 15 items were written again, rephrasing the subject of the assertion, and referring to the one that transcends as the respondent’s community, in a mirror-like version. For example, as of item 11: “Accepting death as a part of life” a second mirror item was built: “Helping others to accept death as part of life”; therefore, a derived scale was constructed, with a reference of the action toward others.

The translation and adaptation of the STS scale by Reed (2009, 2014) was carried out by two English, native speaker teachers and two Spanish fluent students, whose profile coincided with the sample. The proposals were made independently, to later make a consensus agreement.

The first version of the STS, with 15 items, was used to measure the self-transcendence variable, and the second version, with 15 mirrored items, to measure the collective transcendence variable. The items used in the study are presented in Supplementary Annex 1.

The original version of the STS scale proposes a Likert scale of 4 options, with the intention of anchoring the values equidistant and allowing subjective evaluations when scoring (Pena-Gayo et al., 2018). However, in the modified version, the researchers proposed five options, to allow for to indifference or the “I don’t know” answer, which is typical of the population studied (Thiessen, 2017).

Due to the modifications that were made to the STS for the present study, a process of revalidation of the scale was carried out in both versions, personal and collective, before the application. This process is explained below.

### Data Preparation

The database cleaning process was done in two steps, first 10 questionnaires were rejected (2.46% of the total collected) for having a low congruence in their answers, (e.g., questionnaires with a single response value in all items), with which a useful sample of *n* = 392 was obtained. Subsequently, from the total number of useful questionnaires, 60 items without response were identified (0.51% of data absent from the 11,760 expected), to which the imputation criterion was applied by regression from the known data of the items of the respective dimension.

From the correlation matrix, Bartlett’s sphericity test was first applied to analyze the factorization feasibility of the reagents. A positive result was obtained with a significant Chi-square approximation at a *p*-val < 0.000, secondly, the Kaiser-Meyer-Olkin (KMO) test was performed with a result of 0.927, which, being greater than 0.8, also suggests a process of reduction by factorization (Kaiser, 1970; Hair et al., 1999, p. 88–89). Afterward, the unidimensionality of the 30 items in the sample was reviewed from an Exploratory Factor Analysis (EFA) with main components and a Varimax orthogonal rotation (Afifi et al., 2012). When factoring the information, an expected result was obtained, since the items were statistically grouped according to their respective factor by their theoretical construction, 15 for Personal Transcendence (PT) and 15 for Collective Transcendence (CT), where only one item (TP8) pointed to both factors, which is foreseeable due to the nature of the assertion.

### Convergent Analysis and Item Selection

In order to obtain a convergent validity and to be able to carry out the comparative tests between both transcendence constructs, it was decided to only keep the reagents with loads greater than or equal to 0.500 (Kline, 2011), and those that had their respective mirror in both PT and CT factors. The items selected for the analysis, which passed both conditions, were: 2, 4, 5, 7, 9, and 14.

When reducing the information to 12 items, the total variance explained by both factors corresponded to 0.529, which is acceptable according to Hair et al. (1999), who indicate that in social sciences an explained variance less than 60% is completely acceptable. Table 1 shows the factor loadings of the 12 relevant items.

**TABLE 1:** Rotated factor loadings.

IItem	CT factor	PT factor	Commonalities
PT2		–0.796	0.652
PT4		–0.689	0.480
PT5		–0.564	0.429
PT7		–0.520	0.419
PT9		–0.639	0.428
PT14		–0.683	0.502
CT2	0.750		0.610
CT4	0.758		0.612
CT5	0.746		0.574
CT7	0.727		0.546
CT9	0.683		0.55
CT14	0.719		0.547
Variance	3.546	2.804	6.350
% Var	0.296	0.234	0.529

*Varimax rotation; only loadings greater than 0.500 are shown.*

### Internal Consistency

Regarding internal consistency, the PT factor obtained an acceptable Cronbach’s Alpha of 0.771, and the statistical analysis to omit items does not suggest getting rid of one of them (Malhotra et al., 2012; Furr and Bacharach, 2014). The inter-item correlation matrix of the factor (with six items) presents average values between 0.262 and 0.499, and then it is considered valid (Robinson et al., 1991). The CT factor obtained a Cronbach’s alpha of 0.851. Statistical analysis for omitting items does not suggest omitting any. The inter-item correlation matrix of each factor (with six items) presents average values between 0.420 and 0.563, and then it is considered valid.

## Results

### Headings

The average of the items of the Personal Transcendence factor was 4,394 (*DS* = 0.548) and the average of the Collective Transcendence factor was 4,010 (*DS* = 0.721). The descriptive statistics of the responses to each of the items presented in the survey are shown in Table 2. When performing a hypothesis test of mean equality between the items of both dimensions, PT and CT, it turns out that in all of them the responses of the dimension of the PT has a higher or equal rating than the respective dimension of the CT (*p*-val < 0.05).

**TABLE 2:** Answers to: How do I see myself at this moment in my life (*n* = 392)?

PT Items	Average	MSD	CT items	Average	MSD
PT2. Accepting myself as I mature.	4.515**	0.036	CT2. Helping others to accept themselves as they mature.	4.117	0.046
PT4. Adapting to my current life situation.	4.393**	0.042	CT4. Helping others to adjust to their current life situation.	4.028	0.048
PT5. Adjusting to changes in my physical and intellectual abilities.	4.230**	0.042	CT5. Helping others adjust to changes in their physical and intellectual abilities.	3.860	0.049
PT7. Finding meaning in my experiences.	4.391**	0.042	CT7. Helping others to find meaning in their experiences.	3.875	0.050
PT9. Learning from others.	4.503**	0.037	CT9. Motivating people to continue learning from others.	4.171	0.045
PT14. Enjoying my rhythm of life.	4.334**	0.044	CT14. Helping others to enjoy their rhythm of life.	4.010	0.050

Differential: 1, Strongly disagree a 5, Totally agree. MSD, Mean Standard Deviation. **Significant difference with p-val < 0.050.

To evaluate the model and test the research hypotheses, the information was first analyzed from a discriminant validity test and later a Confirmatory Factor Analysis (CFA).

### Discriminant Validity

The correlation between both PT and CT factors is 0.524 with a confidence interval between 0.449 and 0.593 at 95%. Now, to check the discriminant validity, following the method proposed by Fornell and Larcker (1981), if the square root of the AVE of each factor is greater than the inter-factor correlation, then there is a discriminant validity. In the case of the study, the square root of the AVE for the PT factor is 0.654 and for the CT 0.731, both higher than the interfactorial correlation of 0.524 (*p*-val < 0.05); therefore, the results obtained from the sample selected, indicate that there is a significant discriminant validity between the two PT and CT analyzed factors.

### Confirmatory Factor Analysis

The two-dimensional theoretical model PT and PT was analyzed by the MLE (Maximum Likelihood Estimation) method, which reflects in the results of the analysis of the estimated and observed covariate matrices (Kline, 2011). When verifying the adjustment measures, it is observed that the result of the adjustment measures TLI = 0.907 and CFI = 0.944 was satisfactory (higher than 0.90). The RMSEA (0.053) also fell within the recommended parameters, that is, values from 0.05 to 0.08 are acceptable (Hair et al., 1999; Kline, 2011). The remaining measures GFI = 0.990; AGFI = 0.985 and NFI = 0.930 are above the recommended reference value, which is 0.90 (Byrne, 2010; Kline, 2011; Bagozzi and Yi, 2012). The t-val analysis of the loadings of all the items of the two factors, it turns out that none is less than 1.96, which shows the significance of the model in particular. When analyzing the standardized solution according to Hair et al. (1999, p. 605), obtained from the sample of 392 observations, it is observed that all the factorial loadings of the items with their respective latent, PT and CT, are greater than 0.50. The R2 of the estimators of the individual structural equations of the items (Maximum Likelihood) suggest not rejecting any item, since all are greater than 0.25.

Based on the results of the discriminant analysis and the CFA, hypothesis H1 is approved: The orientation toward PT and orientation toward CT are differentiable categories.

### Hierarchy of the Two Factors

To determine in which factor the young people surveyed spend more time (based on the question Do I see myself at this moment in my life?), a mean difference test was used. The statistics of both factors are (*n* = 392): of the average of PT items = 4.411 (DSM = 0.027); of the average of CT items = 3.961 (DSM = 0.035). The difference in means between the PT factor and the CT factor is 0.451 in favor of the PT, with a 0.05 level of significance. The confidence interval (95% certainty) of the difference between both means ranges between 0.365 and 0.537, much higher than zero, which proves that respondents see themselves more in a personal hierarchy than a collective one, in terms of transcendence. With the above, hypothesis H2 is approved: Young people prefer to seek a personal transcendence rather than a collective one, at least for the segment of those who participated in the sample.

An unplanned result of the research was obtained from the regression analysis between CT and PT, in which a positive and significant association is found between both variables.


CT= 0.981+ 0.6894⁢PT+e

With a *t*-val = 12.15, with *p*-val < 0.001 (with a 95% confidence interval for the coefficient between 0.578 and 0.801), and with an adjusted R2 coefficient = 27.29%, which suggests that the higher the PT, the greater the probability of also presenting an orientation toward CT, and vice versa.

## Discussion

Few topics have generated as much literature as Maslow’s hierarchies of human needs. He himself foresaw that self-actualization would be a challenging topic for research, both for promoters and detractors, which makes it a current and exciting topic, especially before the new generations. The discussion between pursuing an individual actualization or a collective one has now gained strength, especially from the hyperconnectivity in which young people live. There are very serious studies that show that individualism is on the rise (e.g., longitudinal ones that span more than 50 years of history); however, there are also studies that determine that this trend depends on the cultural group in question. Faced with this scenario, two questions were proposed: if there is enough statistical evidence to determine that Maslow’s last transcendence level can be divided into two: personal transcendence and collective transcendence. If this is the case, is personal trascendence prior to collective transcendence?

The first finding of the present research, based on the discriminant analysis and the CFA, is that personal transcendence and collective transcendence are differentiable categories, which complements the studies by Maslow (1969b), Koltko-Rivera (1998), Becker et al. (2012), Becker et al. (2014), and Wong (2016), at least within the segment of young people. There is a discriminating level between the dimension of personal transcendence and collective transcendence; therefore, it is concluded that the dimension of personal transcendence is different from the dimension of collective transcendence, where the self is immersed in both postulates, but as a different beneficiary. With this result, it is possible to reflect on the context of social exchanges related to collective transcendent values (Zumeta et al., 2020) of the subjects in search of their happiness, and to reafirm a positive vision for life education and society (Wong, 2016).

The second finding was obtained when realizing that all the motivational acts of the young people surveyed had a statistically higher individual connotation than the collective one (6 out of 6). In other words, young people are preferably looking for a personal self-transcendence instead of a collective one. In this sense, Serrata Malfitano et al. (2019) suggest that collective transcendence does not necessarily happen in everyday life. Rather, as Zumeta et al. (2020) point out, for a person to experience collective transcendence, the existence of triggers, rituals and collective actions is needed.

**FIGURE 1:**
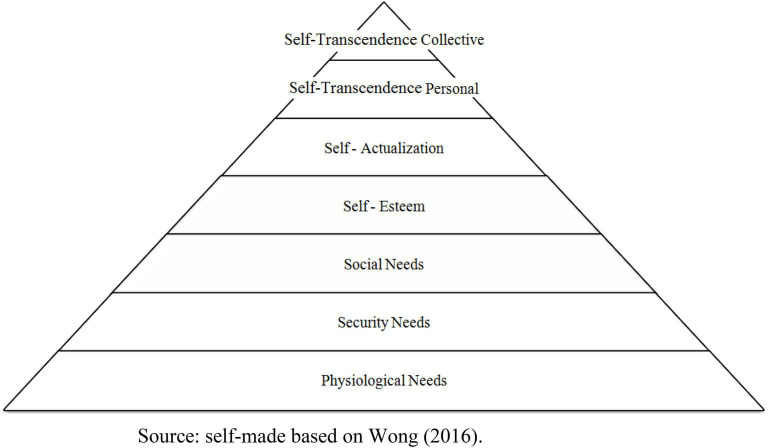
Maslow’s modified extended hierarchy of needs including spiritual needs. The self-transcendence level is divided by personal and collective.

In order to illustrate the extended theory of Maslow, who already places self-transcendence as the highest human goal/achievement above self-actualization, an illustration of how the hierarchies of transcendence is presented in Figure 1, distinguishing the identified factors of personal and collective transcendence.

## Conclusion

In conclusion, young people spend more time in search of a personal transcendence instead of a collective transcendence, which triggers a new series of questions for future research: Is this only happening in Latin America? Alternatively, as dictated by the Latin principle “No one gives what he does not have,” if for a matter of planned order, do young people first seek their transcendence and then help others? On the other hand, this effect is the result of a moral egocentric order, such as it is proposed by Michel Foucault in his ethics of caring for oneself as a practice of freedom: “the care of the self is ethically prior (to care for others) in that relationship with oneself is ontologically prior” (Foucault, 2009, p. 1). The question why young people spend more time in self-transcendence instead of collective transcendence remains open. The answer is certainly relevant to parents, teachers, employers and governments. Differentiating and prioritizing personal and collective transcendence has implications in the way a community is built and the different areas in which its members are involved.

One of the limitations of the present study is that, in order to ensure confidentiality, no demographic data was requested. Likewise, the sample includes only young people, middle-class university students. However, the strength of the study is in proposing a scale of transcendence from a community perspective, from the point of view of the other, and as a second strength, the beginning of the discussion is presented on whether any of the transcendence proposed by Maslow precedes to the other. In future research, the opportunity is presented to review the collective transcendence against other constructs of the human personality (such as religious orientation, political participation or academic vocation of a social nature, such as a teacher or a doctor) and the possibility to apply the scale of collective transcendence in other cultures that are essentially more or less collective.

## Data Availability Statement

The original contributions presented in the study are included in the article/Supplementary Material, further inquiries can be directed to the corresponding author/s.

## Ethics Statement

Ethical review and approval was not required for the study on human participants in accordance with the local legislation and institutional requirements. The patients/participants provided their written informed consent to participate in this study.

## Author Contributions

LFL elaborated the study design, its implementation, and wrote the first draft of the manuscript. LMV contributed to the conception, wrote sections of the manuscript, and revised the manuscript. Both authors read and approved the submitted version.

## Conflict of Interest

The authors declare that the research was conducted in the absence of any commercial or financial relationships that could be construed as a potential conflict of interest.

## Publisher’s Note

All claims expressed in this article are solely those of the authors and do not necessarily represent those of their affiliated organizations, or those of the publisher, the editors and the reviewers. Any product that may be evaluated in this article, or claim that may be made by its manufacturer, is not guaranteed or endorsed by the publisher.
