# Supplementary material for: From Self-Transcendence to Collective Transcendence: In Search of the Order of Hierarchies in Maslow’s Transcendence
Source: Front Psychol. 2022 Mar 24;13:787591. doi: 10.3389/fpsyg.2022.787591 (PMC8988189; doi:10.3389/fpsyg.2022.787591)
Supplement: Supplementary file 1 [file Table_1.docx]

**Annex 1. Applied ítems in Spanish and English**

| **TP** | **Ítems aplicados para la Trascendencia Personal** | **TC** | **Ítems aplicados para la Trascendencia Colectiva** | **Selección de Ítems para el estudio** |
| --- | --- | --- | --- | --- |
| TP1 | Disfrutando pasatiempos y entretenimientos. | TC1 | Desarrollando pasatiempos y entretenimientos para que otros los disfruten. |  |
| TP2 | Aceptándome a mí mismo a medida que maduro. | TC2 | Ayudando a los demás a aceptarse a sí mismos a medida que maduran. | Si |
| TP3 | Participando con mi comunidad | TC3 | Invitando a otras personas a participar con la comunidad. |  |
| TP4 | Adaptándome a mi actual situación de vida | TC4 | Ayudando a los demás a que se adapten a su actual situación de vida. | Si |
| TP5 | Ajustándome a los cambios de mis capacidades físicas e intelectuales. | TC5 | Ayudando a otros a ajustarse a los cambios de sus capacidades físicas e intelectuales. | Si |
| TP6 | Compartiendo mi experiencia con otros. | TC6 | Proponiendo a otros que compartan su experiencia. |  |
| TP7 | Encontrándole significado a mis experiencias. | TC7 | Buscando que otros encuentren un significado a sus experiencias. | Si |
| TP8 | Ayudando a otras personas. | TC8 | Promoviendo que otros ayuden a otras personas. |  |
| TP9 | Aprendiendo de otros. | TC9 | Motivando a otros a seguir aprendiendo de los demás. | Si |
| TP10 | Dejando cosas que alguna vez pensé que eran importantes. | TC10 | Ayudando a los demás a dejar algunas cosas que alguna vez pensaron que eran importantes. |  |
| TP11 | Aceptando la muerte como parte de la vida. | TC11 | Ayudando a los demás a aceptar la muerte como parte de la vida. |  |
| TP12 | Encontrándole significado a mis creencias espirituales. | TC12 | Ayudando a los demás a encontrar significado en sus creencias espirituales. |  |
| TP13 | Permitiendo que otros me ayuden cuando lo necesito. | TC13 | Promoviendo a que otros ayuden cuando alguien lo necesita. |  |
| TP14 | Disfrutando mi ritmo de vida. | TC14 | Buscando que otros disfruten su ritmo de vida. | Si |
| TP15 | Dándole vueltas a mis sueños que no he alcanzado. | TC15 | Apoyando a otros a que alcancen sueños. |  |

| **PT** | **Items applied to personal Trascendence** | **CT** | **Items applied to collective trascendence** | **Selected Items for the study** |
| --- | --- | --- | --- | --- |
| PT1 | Enjoying hobbies and entertainment | CT1 | Developing hobbies and entertainment for others to enjoy. |  |
| PT2 | Accepting myself as I mature | CT2 | Inviting other people to participate with the community. | YES |
| PT3 | Participating with my community | CT3 | Inviting other people to participate with the community. |  |
| PT4 | Adapting to my current life situation | CT4 | Helping others to adapt to their current life situation | YES |
| PT5 | Adjusting to changes in my physical and intellectual abilities. | CT5 | Helping others to adjust to changes in their physical and intellectual abilities | YES |
| PT6 | Sharing my experience with others. | CT6 | Proposing others to share their experience. |  |
| PT7 | Finding meaning in my experiences | CT7 | Looking for others to find meaning in their experiences | YES |
| PT8 | Helping other people. | CT8 | Promoting others to help other people. |  |
| PT9 | Learning from others. | CT9 | Motivating others to continue learning from others | YES |
| PT10 | Leaving things that I once thought were important | CT10 | Helping others to put down some things they once thought were important. |  |
| PT11 | Accepting death as part of life | CT11 | Helping others to accept death as part of life. |  |
| PT12 | Finding meaning in my spiritual beliefs | CT12 | Helping others to find meaning in their spiritual beliefs. |  |
| PT13 | Allowing others to help me when I need it | CT13 | Promoting others to help when someone needs it. |  |
| PT14 | Enjoying my rhythm of life. | CT14 | Looking for others to enjoy their rhythm of life. | YES |
| PT15 | Thinking about my dreams that I have not reached. | CT15 | Supporting others to achieve dreams |  |
